# Emergence and Epidemiology of Ciguatera in the Coastal Cities of Southern China

**DOI:** 10.3390/md13031175

**Published:** 2015-03-02

**Authors:** Thomas Y. K. Chan

**Affiliations:** 1 Division of Clinical Pharmacology and Drug and Poisons Information Bureau, Department of Medicine and Therapeutics, Faculty of Medicine, The Chinese University of Hong Kong, Prince of Wales Hospital, Shatin, New Territories, Hong Kong, China; E-Mail: tykchan@cuhk.edu.hk; Tel.: +852-2632-3907; Fax: +852-2646-8756; 2 Centre for Food and Drug Safety, Faculty of Medicine, The Chinese University of Hong Kong, Hong Kong, China

**Keywords:** ciguatera, ciguatoxins, China, tiger grouper

## Abstract

In the present review of 23 published case studies, the main objective is to report the emergence and epidemiology of ciguatera in the coastal cities of southern China. There was a sudden surge in ciguatera outbreaks in 2004. Ciguatera mostly occurred in the Guangdong Province. In Shenzhen, the incidence of ciguatera in 2004 was estimated to be over 7.5 per million people. In Foshan and Zhongshan, three large outbreaks each affecting over 100–200 subjects (caused by tiger grouper served at banquets) accounted for the much higher incidence of ciguatera in 2004 (>48.7 and >129.9 per million people). Humphead wrasse and areolated coral grouper were the other important ciguatoxic fish. In some subjects, risk factors for increased likelihood of (severe) ciguatera were present, namely concomitant alcohol consumption and ingestion of large reef fishes and CTX-rich fish parts. To prevent large outbreaks and severe illness, large apex predators from coral reefs should never be served at banquets and the public should realize the increased risk of severe symptoms due to ingestion of CTX-rich fish parts with alcohol. The systematic collection of accurate details, implementation of risk assessment process and continuing education for the public on prevention are of obvious importance.

## 1. Introduction

Ciguatera results from eating certain tropical and subtropical coral reef fishes that have accumulated ciguatoxins (CTX) in their flesh. These are typically large predatory fishes, including barracuda, Spanish mackerels, groupers, moray eels, snappers and humphead wrasse [1,2,3]. CTX arise through the biotransformation of less polar precursors produced by dinoflagellates in the genus *Gambierdiscus* [4], becoming more potent and progressively concentrated as they pass along the food chain. CTX, which are mainly found in the Pacific (P-CTX), Caribbean (C-CTX) and Indian Ocean (I-CTX) regions, differ in potency (P-CTX > I-CTX > C-CTX) as activators of voltage-sensitive sodium channels [5]. In the Pacific, three major CTX (P-CTX-1, P-CTX-2 and P-CTX-3) exist, with P-CTX-1 (the most potent CTX known) dominating the toxin profiles [5,6]. Ciguatera is characterized by various gastrointestinal, neurological, cardiovascular and general features [1,2,3,5]. Although large variations in the intensity and occurrence of symptoms are observed between subjects, ingestion of a large quantity and the CTX-rich parts (head, viscera, roe and skin) generally cause more severe poisoning and prolonged illness [2,3].

**Figure 1:**
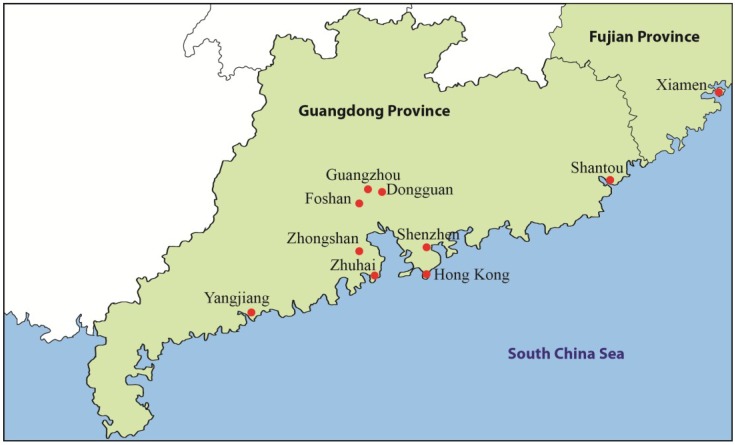
The coastal cities of southern China where ciguatera has been reported.

Ciguatera is frequently reported from non-endemic regions due to increases in international tourism, global trade and consumption of reef fishes from endemic areas [7]. In China (particularly Guangzhou) there is a growing demand for live coral reef fishes and 40%–60% of imports into Hong Kong (the major importer in the world) might be re-exported to the mainland [8]. As demands increase and fish stocks in the neighboring areas become depleted, supplies are increasingly sought from other (or new) fishing grounds [8,9]. In Hong Kong, ciguatera was first reported in the late 1980s [10]. In Shenzhen (located just north of Hong Kong) and other coastal cities of southern China (Figure 1), ciguatera was first reported in 2003 and the 1990s, respectively [11,12]. Reviews on ciguatera in Hong Kong and outbreaks after consumption of brown marbled grouper (tiger grouper) (*Epinephelus fuscoguttatus*) and humphead wrasse (*Cheilinus undulatus*) have recently been published [2,3,13]. In the present review of published case studies, the main objective is to report the emergence and epidemiology of ciguatera in the coastal cities of southern China.

## 2. Published Case Series of Ciguatera

To identify relevant papers in indexed medical journals and Chinese medical journals, a search of Medline (1980 to 29 November 2014) and China Journal Jet (1994 to November 2014) was performed, using ciguatera and ciguatoxins as the search terms. Redundant publications reporting substantially the same case series, news briefs and the only report of ciguatera from outside southern China (2 subjects in Dalian, Liaoning Province after eating humphead wrasse) [14] were excluded.

There was a report from Xiamen, Fujian Province [15]. All other reports were from the Guangdong Province–Dongguan [16], Foshan [17,18,19,20,21], Guangzhou [22], Shantou [23,24,25,26], Shenzhen [11,27,28,29,30], Yangjiang [31], Zhongshan [12,32,33] and Zhuhai [34,35] (see Table 1). Three reports [15,27,30] were from the local health authorities in Xiamen [15] and Shenzhen [27,30]. All other reports were hospital-based case series from: 1 hospital each in Dongguan, Guangzhou, Shantou and Yangjiang, 2 hospitals in Foshan and Zhuhai, and 3 hospitals in Shenzhen and Zhongshan. Since the clinical presentations of ciguatera in the Pacific region in general [5,7,13] and features specific to tiger grouper and humphead wrasse [2,3] are now well recognized, only the epidemiological features and possible risk factors in the coastal cities of southern China are summarized here. Details on symptoms after consumption of tiger grouper [15,19,26,33] and humphead wrasse [11,28,29] were available elsewhere [2,3].

The 23 reports covered the 15-year period between 1994 and 2008 (Table 1). There were 14 reports in 2004; 9 other reports were from 1994–2003 (*n =* 1), 1998–1999 (*n =* 1), 1999 (*n =* 1), 2003–2004 (*n =* 1), 2004–2006 (*n =* 1), 2005 (*n =* 2), 2005–2006 (*n =* 1) and 2005–2008 (*n =* 1). All 23 reports were brief, with variable amounts of details about the epidemiological and clinical features.

The number of subjects involved in each outbreak was mentioned in 16 reports (see Table 1). Three reports were from large outbreaks in 2004 affecting over 100–200 subjects who had eaten tiger grouper served at banquets [19,32,33]. In 13 other reports, the number of subjects in each outbreak varied from 2 to 64. Children under 12, and as young as 1.4, were involved.

The annual incidence of ciguatera could be estimated. In Shenzhen, if the city-wide figures from the local health authorities were used [27], the incidence would be 7.5 per million people in 2004. Under-reporting was obvious (Table 1) since hospital-based case series [11,28,29] indicated a greater number of outbreaks, subjects affected and fish species involved. In 2005–2006, based on the official data [30], the mean incidence was 1.1 per million people. In Foshan and Zhongshan, based on the published case series in 2004 [17,18,19,20,32,33], the incidence was >48.7 and >129.9 per million people, respectively.

The fish species responsible for these ciguatera outbreaks were stated in 20 reports (Table 1). Tiger grouper, humphead wrasse and leopard coral grouper (*Plectropomus leopardus*) were involved in 11, 8 and 5 reports, respectively. Sea bass and flowery grouper (*Epinephelus polyphekadion*) were involved in 2 reports. Areolated coral grouper (*Plectropomus areolatus*), moral eel (*Gymnothorax monochrous*) and giant grouper (*Epinephelus lanceolatus*) were involved in 1 report. The fish species involved were not specified in 3 reports–grouper in 2 and reef fish in 1.

**Table 1:** Outbreaks of ciguatera in the coastal cities of southern China.

City (Reference)	Period	Sex	Age (year) ^d^	Details
Xiamen				
[15] ^a^	2/2005	29M/F ^c^	(9–66)	3 outbreaks (*n =* 6–11 in each), after eating portions of large tiger grouper, fish size reflected by the weight of left-over portions (~5 kg), 16 subjects hospitalized
Dongguan				
[16]	10/2004	6M7F	(23–66)	1 outbreak (*n =* 14), after sharing a reef fish, 13 subjects admitted to hospital after eating the flesh with skin plus fish head (*n =* 4), fish belly (*n =* 8) or fish viscera (*n =* 4), 1 subject who ate only the flesh had minimal symptoms
Foshan				
[17]	2004	20M/F ^c^	- ^c^	1 outbreak (*n =* 20), after sharing a grouper (11.5 kg), 2 subjects hospitalized in cardiac center
[18]	11/2004	12M29F	42(21–92)	1 outbreak (*n =* 41), after eating giant grouper, all 41 subjects hospitalized
[19] ^a^	11/2004	17M27F	(11–64)	1 outbreak (*n* > 100), after eating tiger grouper in a banquet, 44 subjects admitted to this hospital
[20]	11/2004	1M1F	(44–47)	1 outbreak (*n* > 10), after sharing a grouper, 2 subjects admitted to this hospital
[21]	1/2004–12/2006	16M26F	41(11–60)	3 outbreaks (*n =* 42), after eating tiger grouper or leopard coral grouper, all 42 subjects hospitalized
Guangzhou				
[22]	1–4/1999	4M5F	45(5–80)	9 subjects hospitalized, after eating moray eel (flesh or viscera)
Shantou				
[23]	3/1998–4/1999	18M7F	(1.4–58)	6 (4 in 1998, 2 in 1999) outbreaks (*n =* 3–6 in each), after eating tiger grouper (*n =* 4) or sea bass (*n =* 2), 25 subjects hospitalized
[24]	6/2000–12/2004	61M21F	(3–68)	82 subjects hospitalized, after eating humphead wrasse, tiger grouper, flowery grouper, areolated coral grouper, *etc.*
[25,26] ^a^	8/2004	48M16F	(4–76)	1 outbreak (*n =* 64), after sharing 2 tiger groupers (>7 kg each), all 64 subjects hospitalized, concomitant alcohol consumption (23 out of 59 subjects) increased the risk of bradycardia (78% *vs.* 19%), hypotension (48% *vs.* 14%) and altered skin sensation (96% *vs.* 42%)
Shenzhen				
[11] ^b^	10/2003–10/2004	14M18F	45(12–68)	32 subjects hospitalized (*n =* 2–16 in each outbreak), after eating humphead wrasse
[27]	1–12/2004	- ^c^	- ^c^	4 outbreaks, 60 subjects affected, caused by humphead wrasse (*n =* 3) or leopard coral grouper (*n =* 1)
[28] ^b^	10/2004	18M8F	46(21–62)	26 subjects hospitalized, after sharing a humphead wrasse (14.5 kg)
[29] ^b^	10/2004	24M15F	(2–78)	7 outbreaks (*n =* 3–12 in each), after eating humphead wrasse
[30]	1/2005–12/2006	- ^c^	- ^c^	3 outbreaks, 36 subjects affected, caused by humphead wrasse (*n =* 2) or leopard coral grouper (*n =* 1)
Yangjiang				
[31]	4/2005–12/2008	10M7F	30(13–60)	4 outbreaks, caused by humphead wrasse, leopard coral grouper or tiger grouper, size 2.0–4.3 kg, 17 subjects with cardiovascular features hospitalized
Zhongshan				
[12]	4/1994–12/2003	33M53F	38(8–87)	86 out of 358 subjects with cardiovascular features, after eating tiger grouper, leopard coral grouper or sea bass, size 1.5–2.5 kg, concomitant alcohol consumption in 85% of males
[32]	11/2004	54M78F	43	1 outbreak, after eating reef fish (tiger grouper ^e^) in a banquet, 132 subjects admitted to this hospital
[33] ^a^	11/2004	36M27F	43(23–70)	1 outbreak (*n* > 200), after eating tiger grouper in a banquet, 63 subjects admitted to this hospital
Zhuhai				
[34]	5–7/2004	9M6F	42(26–73)	15 subjects hospitalized, after eating flowery grouper (*n =* 10) or grouper (*n =* 5)
[35]	6/2005	1M1F	(36–41)	2 subjects hospitalized, after eating humphead wrasse (flesh and viscera)

Refer to references ^a^ [2] and ^b^ [3] for details on signs and symptoms of ciguatera; All were hospital-based studies, except reports [15], [27] and [30] from the local health authorities with incomplete data; ^c^ The sex or age distribution was not stated; ^d^ Age as mean (range), except for median (range) for report [12]; ^e^The fish species involved was not specified, but according to [33], it was tiger grouper.

The size of the fish involved was described in six reports. In Xiamen, the left-over portions of a tiger grouper causing three outbreaks weighed ~5 kg [15]. In Foshan, a grouper (11.5 kg) was responsible for an outbreak [17]. In Shantou, two tiger groupers (>7 kg) were responsible for a large outbreak [25,26]. In Shenzhen, a humphead wrasse (14.5 kg) caused an outbreak [28]. In Yangjiang, four outbreaks occurred after ingestion of humphead wrasse, leopard coral grouper and tiger grouper weighing 2.0–4.3 kg [31]. In Zhongshan, tiger grouper, leopard coral grouper and sea bass weighing 1.5–2.5 kg were responsible for outbreaks in 1994–2003 [12].

Factors associated with increased risk of (severe) ciguatera [2,3] were observed in some subjects, including concomitant alcohol consumption [25,26] and ingestion of large reef fishes [15,17,25,26,28] and CTX-rich fish parts [16,22,35].

## 3. Discussion

The present review of 23 published reports (see Table 1) helps define the epidemiology of ciguatera in the coastal cities of southern China. The yearly number of reports indicated that there was a sudden surge in ciguatera outbreaks in 2004 (*n =* 14). There were far fewer reports in 1994–2003 (*n =* 3), 2003–2004 (*n =* 1), 2004–2006 (*n =* 1) and 2005–2008 (*n =* 4). The geographical distribution of reports indicated that ciguatera mostly occurred in the Guangdong Province (*n =* 22). In the recent 2 decades, with better living standards and the economic growth, there was a growing demand for reef fish, especially among residents of the southeast coast [12]. The fish could originate from South China Sea [36] and other reef areas via Hong Kong [8]. If fish stocks in the neighboring regions become depleted, supplies might be increasingly sought from other (or new) fishing grounds [8,9].

Under-reporting of ciguatera is common, mainly because of non-reporting and misdiagnosis of mild cases as other more common illnesses [1]. In Shenzhen, hospital-based case series [11,28,29] indicated a greater number of outbreaks, subjects affected and fish species involved than the annual figures from the local health authorities [27]. Thus, the actual incidence of ciguatera in 2004 should be over 7.5 per million people. In contrast, large outbreaks affecting over 100–200 subjects, as seen in 2004 in Foshan [19] and Zhongshan [32,33], would be widely publicized. These 3 large outbreaks explained the much higher incidence of ciguatera (>48.7 and >129.9 per million people) there. Unfortunately, the reporting mechanisms for ciguatera and the data for sporadic cases and milder cases not requiring hospitalization were not known. The systematic collection of accurate details and implementation of risk assessment process [37] are of obvious importance.

The predominant reef fish species associated with ciguatera vary with the geographical regions [1]. In the coastal cities of southern China, the groupers were the most important cause of ciguatera (Table 1). In particular, the tiger grouper was responsible for three large outbreaks in 2004 [19,32,33]. Humphead wrasse was also an important ciguatoxic fish. Both fishes are much sought-after, high-valued species in the live reef food-fish trade. As expected of the apex predators from coral reefs of the Indo-Pacific region, both fishes are likely to be ciguatoxic [2,3]. Although their ciguatoxic potential is expected to be fish size-dependent, the length threshold above which the risk of ciguatera significantly increases is not easy to define [2,3], because of lack of research data and regional variations in findings.

Outbreaks of ciguatera fish poisoning can occur at home or food premises. Exposures to ciguatoxic fish (tiger grouper) served at banquets were the reason for the 3 large outbreaks in 2004 in Foshan [19] and Zhongshan [32,33]. The size of fish involved and proportion of subjects with concomitant alcohol ingestion were not mentioned. To prevent large outbreaks and severe illness, large apex predators from coral reefs should never be served at banquets and the public should realize the increased risk of severe symptoms due to concomitant alcohol consumption [2,3]. For outbreaks that occurred in other settings, risk factors for increased likelihood of (severe) ciguatera [2,3] were seen in some subjects, namely concomitant alcohol consumption and ingestion of large reef fishes (>7 to 14.5 kg) and CTX-rich fish parts (head and viscera). Continuing education for the public on prevention of ciguatera was required.

It would be great interest to compare the epidemiology of ciguatera in the coastal cities of southern China and Hong Kong (Table 2).

**Table 2:** Epidemiology of ciguatera in the coastal cities of southern China and Hong Kong.

	Southern China 1994–2008	Hong Kong [13] 1989–2008
Incidence/million people (year)	1.1 (2005/6) ^a^ to 7.5 (2004) ^a^	3.3 to 64.9 (median 10.2)
	>48.7 (2004) ^b^	1st peak–64.9 (1998)
	>129.9 (2004) ^c^	2nd peak–35.5 (2004)
Large outbreaks (>100–200 subjects)	3	0
Fish species causing large outbreaks	Tiger grouper	–
Important fish species ^d^	Tiger grouper, humphead wrasse, areolated coral grouper	Snappers (until 1996)
		Groupers (from 1997) ^d^

Based on city-wide figures in ^a^ Shenzhen and hospital-based case series in ^b^ Foshan and ^c^ Zhongshan; ^d^ Groupers (tiger grouper, leopard coral grouper, lyretail grouper, flowery grouper, spotted coral grouper), moray eel, two-spot red snapper and humphead wrasse, *etc.* were commonly involved [13,38].

In the coastal cities of southern China and Hong Kong, there was a growing demand for reef fishes. They had similar sources of reef fishes [8,36,39], but the epidemiology of ciguatera was quite different (Table 2). In particular, there were 3 large outbreaks in southern China (caused by tiger grouper), but none in Hong Kong, accounting for the higher incidence of ciguatera in Foshan and Zhongshan.

## 4. Conclusions

The present review of 23 published reports helps define the epidemiology of ciguatera in the coastal cities of southern China. The yearly number of reports indicated a sudden surge in ciguatera outbreaks in 2004. The geographical distribution of reports had confirmed that ciguatera mostly occurred in the Guangdong Province. In Shenzhen, the incidence of ciguatera in 2004 should be over 7.5 per million people. In Foshan and Zhongshan, three large outbreaks affecting over 100–200 subjects (caused by tiger grouper served at banquets) accounted for the much higher incidence of ciguatera in 2004 (>48.7 and >129.9 per million people, respectively). Humphead wrasse and areolated coral grouper were the other important ciguatoxic fish. Risk factors for increased likelihood of (severe) ciguatera were seen in some subjects–concomitant alcohol consumption, ingestion of large reef fishes (>7 to 14.5 kg) and CTX-rich fish parts (head and viscera). To prevent large outbreaks and severe illness, large apex predators from coral reefs should never be served at banquets and the public should realize the increased risk of severe symptoms due to ingestion of CTX-rich fish parts with alcohol. The systematic collection of accurate details, implementation of risk assessment process and continuing education for the public on prevention are of obvious importance.
